# Evidências de validade da *Escala de Satisfação com a Assistência Hospitalar ao Parto* utilizando a Teoria da Resposta ao Item

**DOI:** 10.1590/0102-311XPT026525

**Published:** 2026-02-16

**Authors:** Camila Bonalume Dall’Aqua, Stela Maris de Jezus Castro, Betine Pinto Moehlecke Iser, Janini Cristina Paiz, Antônio Augusto Moura da Silva, Dayana Dourado de Oliveira Costa, Maria do Carmo Leal, Camila Giugliani

**Affiliations:** 1 Faculdade de Medicina, Universidade Federal do Rio Grande do Sul, Porto Alegre, Brasil.; 2 Programa de Pós-graduação em Ciências da Saúde, Universidade do Sul de Santa Catarina, Tubarão, Brasil.; 3 Departamento de Saúde Pública, Universidade Federal do Maranhão, São Luís, Brasil.; 4 Escola Nacional de Saúde Pública Sergio Arouca, Fundação Oswaldo Cruz, Rio de Janeiro, Brasil.

**Keywords:** Psicometria, Satisfação do Paciente, Estudos de Validação, Saúde Materna, Parto, Psychometrics, Pacient Satisfaction, Validation Studies, Maternal Health, Parturition, Psicometría, Satisfación del Paciente, Estudios de Validación, Salud Materna, Parto

## Abstract

Com o objetivo de produzir evidências de validade sobre uma medida para o traço latente “nível de satisfação com o parto”, utilizou-se a Teoria da Resposta ao Item (TRI) em uma amostra de 15.965 mulheres que tiveram parto hospitalar no Brasil. Os dados provêm de entrevistas telefônicas realizadas até seis meses após o parto, com participantes da pesquisa *Nascer no Brasil: Pesquisa Nacional sobre Parto e Nascimento* provenientes das cinco regiões brasileiras. Foi utilizado um questionário de 11 itens denominado *Escala de Satisfação com a Assistência Hospitalar ao Parto*. As questões foram incluídas nos modelos da TRI, sendo o de resposta gradual de Samejima o de melhor ajuste. Dividiu-se aleatoriamente a amostra em: banco 1 (n = 7.982), ajustado pelo modelo de resposta gradual de Samejima; e banco 2 (n = 7.983), aplicando a validação dos resultados. Foram verificadas medidas de fidedignidade: alfa de Cronbach, ômega de McDonald e coeficiente de Spearman-Brown. Na validade de construto, a análise fatorial exploratória do banco 1 demonstrou estrutura fatorial latente com três dimensões. A estrutura resultante foi testada nos dados do banco 2 por análise fatorial confirmatória, obtendo-se bom ajuste do modelo. Dois itens do questionário foram excluídos da composição da medida por não discriminarem o traço latente objeto deste estudo, sendo a Escala finalizada com nove itens. A partir da TRI, apresenta-se uma nova versão do instrumento e conclui-se que o mesmo apresenta propriedades psicométricas adequadas. As associações entre a satisfação com o parto e variáveis externas reforçam as evidências de validade da escala.

## Abstract (en)

With the objective of generating evidence of validity for a measure of the latent trait “level of satisfaction with childbirth”, Item Response Theory (IRT) was used in a sample of 15,965 women who had hospital births in Brazil. Telephone interviews were conducted up to six months after childbirth with participants of the *Birth in Brazil: National Survey into Labor and Birth* in the five regions of the country. An 11-item questionnaire denominated the *Hospital Childbirth Care Satisfaction Scale* was used. The questions were incorporated into the IRT models, with Samejima’s graded response model providing the best fit. The sample was randomly divided into dataset 1 (n = 7,982), adjusted using the graded response model, and dataset 2 (n = 7,983), for validation of the results. Reliability measures were Cronbach’s alpha, McDonald’s omega, and Spearman-Brown coefficient. Regarding construct validity, exploratory factor analysis of dataset 1 demonstrated a latent factor structure with three dimensions. The resulting structure was tested in dataset 2 using confirmatory factor analysis, obtaining a good fit of the model. Two items of the questionnaire were excluded from the measure for not discriminating the latent trait of interest. Thus, thefinal scale consisted of nine items. Based on IRT, a new version of the instrument with adequate psychometric properties was obtained. The associations between satisfaction with childbirth and external variables strengthen the validity of the scale.

## Abstract (es)

Con el objetivo de producir pruebas de validez sobre una medida para el rasgo latente “nivel de satisfacción con el parto”, se utilizó la Teoría de Respuesta al Ítem (TRI) en una muestra de 15.965 mujeres que tuvieron partos hospitalarios en Brasil. Los datos proceden de entrevistas telefónicas realizadas hasta seis meses después del parto a participantes en la encuesta *Nacer en Brasil: Encuesta Nacional sobre Partos y Nascimientos* procedentes de las cinco regiones brasileñas. Se utilizó un cuestionario de 11 preguntas denominado *Escala de Satisfacción con la Asistencia Hospitalaria al Parto*. Las preguntas se incluyeron en los modelos de la TRI, siendo el de respuesta gradual de Samejima el que mejor se ajustó. Se dividió aleatoriamente la muestra en: banco 1 (n = 7.982), ajustado por el modelo de respuesta gradual de Samejima; y banco 2 (n = 7.983), aplicando la validación de los resultados. Se verificaron las medidas de fiabilidad: alfa de Cronbach, omega de McDonald y coeficiente de Spearman-Brown. En cuanto a la validez del constructo, el análisis factorial exploratorio del banco 1 demostró una estructura factorial latente con tres dimensiones. La estructura resultante se probó en los datos del banco 2 mediante un análisis factorial confirmatorio, obteniéndose un buen ajuste del modelo. Dos ítems del cuestionario fueron excluidos de la composición de la medida por no discriminar el rasgo latente objeto de este estudio, quedando la escala finalizada con nueve ítems. A partir de la TRI, se presenta una nueva versión del instrumento y se concluye que este presenta propiedades psicométricas adecuadas. Las asociaciones entre la satisfacción con el parto y las variables externas refuerzan las evidencias de validez de la escala.

## Introdução

A qualidade do atendimento às mulheres no parto pode impactar sua saúde mental e o cuidado de suas crianças ^1.2^. Mulheres expostas a tratamento desrespeitoso no momento do parto parecem ter maior probabilidade de apresentar sintomas de depressão pós-parto ^1^ ^,^ ^3^ ^,^ ^4^ ^,^ ^5^ e desenvolver ansiedade e transtorno do estresse pós-traumático ^6^, enquanto aquelas que demonstram maior satisfação com o atendimento prestado tendem a estabelecer melhor vínculo com o recém-nascido, obtendo maiores taxas de sucesso na amamentação ^7^. Mulheres expostas a maus-tratos no parto tendem a postergar o retorno à unidade de saúde após o nascimento do bebê, implicando a perda de oportunidades de cuidado, tanto para si quanto para seus filhos ^2^.

No Brasil, com a Rede Cegonha (*Portaria nº 1.459*, de 24 de junho de 2011) ^8^, houve grande mudança nos investimentos em saúde materno-infantil no Sistema Único de Saúde (SUS), para garantir atendimento seguro, de qualidade e humanizado. Para aferir as estratégias recomendadas pela Rede Cegonha, previu-se avaliação periódica dos componentes implementados. Nos ciclos avaliativos realizados, um desafio foi a falta de sistemas nacionais que registrassem adequadamente ações de cuidados obstétricos e neonatais prestados pelos serviços de saúde hospitalares. Todavia, a avaliação das maternidades permitiu novos olhares para a assistência obstétrica e neonatal. A reflexão conjunta de trabalhadores e gestores proporcionou aprendizados e oportunidades de repensar a rotina dos serviços ^9^.

Ainda que se discuta sobre limitações da Rede Cegonha em abranger a saúde das mulheres na sua integralidade, sua instituição foi um marco para as políticas de saúde das mulheres no Brasil. Foram desenvolvidas estratégias para qualificação dos serviços, como as *Diretrizes Nacionais de Assistência ao Parto Normal* ^10^ e o *Apice On - Aprimoramento e Inovação no Cuidado e Ensino em Obstetrícia e Neonatologia* ^11^. No âmbito internacional, a Organização Mundial da Saúde (OMS) lançou, em 2018, a publicação *WHO Recommendations: Intrapartum Care for a Positive Childbirth Experience* ^12^. Em setembro de 2024, a *Portaria GM/MS nº 5.350* ^13^ revogou a Rede Cegonha para dispor sobre a Rede Alyne, estratégia que surge para aprimorar a atenção integral à saúde das mulheres no Brasil. Assim, a experiência de parto, aferida pela satisfação das mulheres e pessoas que gestam, vem ganhando espaço nas políticas de saúde e sendo valorizada na avaliação global do cuidado integral em saúde.

Avaliar a satisfação das mulheres em relação aos seus partos exige a escolha de instrumentos adequados ^14^. A participação das parturientes na avaliação do cuidado é essencial para estabelecer indicadores de qualidade e construir melhorias na assistência obstétrica e neonatal ^15^. Muitas são as ações que qualificam a atenção prestada às mulheres no parto, entre elas: boa comunicação com a equipe assistencial, oferecimento de alimentação leve, presença de acompanhante de livre escolha, uso de métodos não farmacológicos para alívio da dor, com incentivo à livre movimentação e escolha da posição para o parto. Contudo, procedimentos como uso contínuo de ocitocina por venóclise durante o trabalho de parto, prática rotineira de episiotomia e amniotomia devem ser extinguidos ^7^ ^,^ ^11^ ^,^ ^12^.

Poucos instrumentos foram desenvolvidos especificamente para avaliar a satisfação com o parto. Alguns estudos mensuraram esse desfecho com uma pergunta direta, tal como “qual a sua satisfação com o atendimento ao seu parto?”, abordagem que se mostrou imprecisa e pouco discriminatória ^7^ ^,^ ^16^. Outros, aplicaram escalas contendo diferentes itens ^17^. Deve-se atentar para os níveis de validade e confiabilidade das escalas, sendo necessários testes para avaliar suas propriedades psicométricas ^14^. Além disso, os itens aplicados em diferentes estudos para medir a satisfação das mulheres com o parto carecem de padronização, o que dificulta a escolha do instrumento mais adequado e o estabelecimento de parâmetros para avaliar aspectos subjetivos da vida das mulheres, ainda pouco explorados ^17^.

No Brasil, a maior pesquisa nacional já realizada no contexto do parto e nascimento - a *Nascer no Brasil* - avaliou, entre 2011 e 2013, a satisfação das usuárias com a assistência hospitalar ao parto, a partir da *Escala de Satisfação com a Assistência Hospitalar ao Parto*. Esse instrumento, contendo 11 itens, foi aplicado por via telefônica com 15.965 mulheres de todas as regiões do Brasil em até seis meses após o parto. A composição do questionário teve como referência perguntas adaptadas da *Pesquisa Mundial de Saúde* (WHS, acrônimo em inglês) ^18^, que mediam a satisfação geral das usuárias com os serviços de saúde; perguntas sobre satisfação com o parto, o puerpério e a assistência neonatal; e uma pergunta direta sobre violência obstétrica (física, psicológica ou verbal). O estudo de suas propriedades psicométricas (consistência interna, associação com variáveis externas e invariância métrica e configural) demonstrou ser um instrumento unidimensional, com evidências de validade na população estudada a partir de uma versão com dez itens, proposta em 2019 ^19^. O presente estudo teve como objetivo produzir novas evidências de validade, utilizando a Teoria da Resposta ao Item (TRI) para estimar o traço latente “nível de satisfação com o parto” na amostra de mulheres da pesquisa *Nascer no Brasil*, a fim de ampliar o espectro de informações psicométricas do instrumento.

## Métodos

### Delineamento, local e população do estudo

Para este estudo, foram utilizados dados secundários da pesquisa de base hospitalar *Nascer no Brasil: Pesquisa Nacional sobre Parto e Nascimento*. Na linha de base, a pesquisa incluiu 23.894 participantes de 266 hospitais públicos e privados, que haviam realizado mais de 500 partos/ano. Em cada hospital, foram convidadas a participar ao menos 90 puérperas e seus recém-nascidos ^20^ ^,^ ^21^. A *Nascer no Brasil* foi desenvolvida em várias etapas e, para este estudo, utilizaram-se os dados das entrevistas realizadas com as mulheres até seis meses após o parto, aplicadas por via telefônica, totalizando uma amostra de 15.965 mulheres ^19^. A amostra do estudo é constituída por delineamento complexo, em que cada estrato assume um peso amostral diferente ^20^ ^,^ ^21^.

### Seleção das participantes e coleta de dados

O seguimento telefônico realizado entre março de 2011 e fevereiro de 2013, por pessoas treinadas, incluiu 66,8% das mulheres da amostra inicial. Para ajustar os pesos amostrais em relação à não resposta, optou-se por modelar as probabilidades de resposta utilizando as informações de covariáveis disponíveis na pesquisa de base ^20^. O questionário continha um instrumento de 11 itens, elaborado para medir o traço latente “nível de satisfação com o parto” (Quadro 1). Tal instrumento teve suas propriedades psicométricas testadas, demonstrando evidências de validade para medir o referido traço latente ^19^. Mais detalhes sobre os métodos da pesquisa *Nascer no Brasil* podem ser consultados em publicações anteriores ^20^ ^,^ ^21^.

**Quadro 1:** Questionário sobre a satisfação das mulheres com a assistência ao parto recebida no hospital. *Nascer no Brasil*, 2011-2013.

QUESTÃO	PERGUNTAS	OPÇÃO DE RESPOSTA
Q1	Na sua internação para o parto, como a senhora avalia o tempo gasto com o deslocamento da sua casa até a maternidade?	1: muito ruim; 2: ruim; 3: moderado; 4: bom; 5: muito bom
Q2	Na sua internação para o parto, como a senhora avalia o tempo de espera desde que chegou na maternidade até ser atendida?	1: muito ruim; 2: ruim; 3: moderado; 4: bom; 5: muito bom
Q3	Na sua internação para o parto, como a senhora avalia o respeito dos profissionais ao recebê-la e ao falar com a senhora?	1: muito ruim; 2: ruim; 3: moderado; 4: bom; 5: muito bom
Q4	Receber um tratamento respeitoso significa ter os exames realizados de maneira respeitosa. Na sua internação para o parto, como a senhora avalia a maneira como sua intimidade foi respeitada durante o exame físico e o atendimento (por exemplo, durante os toques e o atendimento ao parto)?	1: muito ruim; 2: ruim; 3: moderado; 4: bom; 5: muito bom
Q5	Na sua internação para o parto, como a senhora avalia a clareza com que os profissionais de saúde explicaram as coisas para a senhora?	1: muito ruim; 2: ruim; 3: moderado; 4: bom; 5: muito bom
Q6	Na sua internação para o parto, como a senhora avalia o tempo disponível para fazer perguntas sobre a sua saúde ou o seu tratamento?	1: muito ruim; 2: ruim; 3: moderado; 4: bom; 5: muito bom
Q7	Na sua internação para o parto, como a senhora avalia a possibilidade de participar junto com a equipe de saúde das decisões sobre o seu trabalho de parto e o parto?	1: muito ruim; 2: ruim; 3: moderado; 4: bom; 5: muito bom
Q8	Na sua internação para o parto, a senhora considera que foi vítima de algum maltrato ou outra forma de violência por parte dos profissionais?	0: houve violência; 1: não houve violência;
Q9	Na sua opinião, o atendimento ao seu parto foi:	1: muito ruim; 2: ruim; 3: moderado; 4: bom; 5: muito bom
Q10	Na sua opinião, os cuidados e as orientações que a senhora recebeu depois do parto até a alta da maternidade foram:	1: muito ruim; 2: ruim; 3: moderado; 4: bom; 5: muito bom
Q11	Na sua opinião, o atendimento ao bebê na maternidade onde ele nasceu foi:	1: muito ruim; 2: ruim; 3: moderado; 4: bom; 5: muito bom

Fonte: questionário telefônico, puérperas 2011, da pesquisa *Nascer no Brasil* ^19^.

### Análises estatísticas

Para alcançar o objetivo do estudo, foram realizadas as seguintes etapas, que compõem o processo de validação de um instrumento por:

Etapa 1: análise exploratória do banco total (n = 15.965), para avaliação de validade de face dos itens proponentes, bem como verificado o desempenho geral dos itens quando ajustados aos modelos da TRI (resposta gradual de Samejima e crédito parcial generalizado), que permitem avaliar parâmetros de discriminação (ou inclinação) e de dificuldade (ou de posição) para cada item proposto no instrumento de medida ^22^, possibilitando a identificação da necessidade de recategorização das respostas. Os modelos foram ajustados no pacote *mirt*, versão 1.37.1, do R (http://www.r-project.org). Para a escolha do modelo, utilizaram-se as medidas de qualidade de ajuste AIC (critério de informação de Akaike), AICc (critério de informação de Akaike corrigido), SABIC (critério de informação Bayesiano ajustado pelo tamanho da amostra) e BIC (critério de informação Bayesiano). A suposição de unidimensionalidade suficiente dos modelos da TRI considera que o primeiro fator latente explique pelo menos 20% da variação total dos itens ^23^ ^,^ ^24^ ^,^ ^25^ ^,^ ^26^. Neste estudo, esta suposição foi verificada por meio da análise fatorial exploratória utilizando o pacote *psych*, versão 2.2.9, do R.

**Figura 1:**
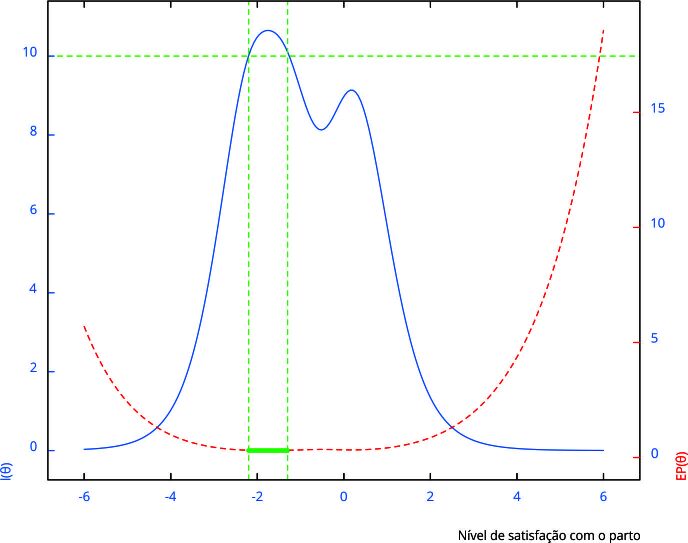
Curva de informação e erro padrão (EP) da *Escala de Satisfação com a Assistência Hospitalar ao Parto*.

Etapa 2: divisão aleatória do banco de dados em duas partes. O banco 1 (n = 7.982) foi ajustado ao modelo de resposta gradual de Samejima (modelo que apresentou melhor desempenho na etapa 1) ^22^. O cálculo da probabilidade de uma mulher escolher a categoria *k* ou maior que *k*, do item *i*, encontra-se no Material Suplementar (Apêndice 1; https://cadernos.ensp.fiocruz.br/static//arquivo/supl-e00026525_6256.pdf). Os parâmetros do modelo referentes aos itens do instrumento são: discriminação do item e dificuldade de cada categoria de resposta. O ajuste do modelo pode ser visualizado nas curvas de categoria de resposta do item (Material Suplementar - Figura S1; https://cadernos.ensp.fiocruz.br/static//arquivo/supl-e00026525_6256.pdf), de informação do item (Material Suplementar - Figura S2; https://cadernos.ensp.fiocruz.br/static//arquivo/supl-e00026525_6256.pdf) e de informação e erro padrão da *Escala de Satisfação com a Assistência Hospitalar ao Parto* (Figura 1). O escore (nível de satisfação com o parto) deste modelo tem escala com média 0 e desvio padrão (DP) 1. Com o banco 2 (n = 7.983), foram estimados níveis de satisfação das mulheres com o parto, utilizando as estimativas dos parâmetros do modelo de resposta gradual de Samejima ^22^ obtidos com o ajuste no banco 1.

Etapa 3: a precisão do escore do nível de satisfação com o parto é apresentada na Figura 1. Como informação complementar, foram estimadas as medidas de fidedignidade alfa de Cronbach, ômega de McDonald (ambas com intervalo de 95% de confiança - IC95% - obtido por *Bootstrap*), utilizando os pacotes *ltm*, versão 1.2-0, e *coefficientalpha*, versão 0.7, respectivamente, e o coeficiente de Spearman-Brown, a partir do método das metades, nos bancos 1 e 2 ^27^ ^,^ ^28^.

Etapa 4: para validade de construto, realizou-se a análise fatorial exploratória (AFE) no banco 1 (n = 7.982), a fim de verificar quantos construtos comuns são necessários para explicar as intercorrelações entre os itens (estrutura fatorial latente) ^27^. A AFE baseou-se na matriz de correlações policóricas dos itens politômicos e utilizou-se rotação oblíqua Promax. Uma medida da adequabilidade dos dados ao modelo de análise fatorial é o Kaiser-Meyer-Olkin (KMO), que pode variar de 0 a 1, sendo aceitável KMO > 0,5 ^29^ ^,^ ^30^ ^,^ ^31^. Posteriormente, foi realizada uma análise fatorial confirmatória (AFC) no banco 2 (n = 7.983), com medidas de ajuste como quadrado médio comparativo (CFI > 0,95 indica bom ajuste), índice Tucker Lewis (TLI > 0,97 indica bom ajuste), raiz do erro quadrático médio de aproximação (RMSEA < 0,05 indica bom ajuste) e raiz quadrada média residual padronizada (SRMR < 0,05 indica bom ajuste), para avaliar se a estrutura fatorial latente encontrada no banco 1 se ajustava aos dados do banco 2 ^32^ ^,^ ^33^ ^,^ ^34^ ^,^ ^35^ ^,^ ^36^. A AFE utilizou o pacote *psych*, versão 2.2.9, e a AFC o pacote *lavaan*, versão 0.6-13 do R.

Etapa 5: quanto à validade concorrente (validade de critério) - evidência de que o escore obtido pelo modelo de Samejima de fato mede o nível de satisfação com o parto ^27^ -, foram verificadas associações brutas e ajustadas do referido traço com características das mulheres sabidamente relacionadas à satisfação com o parto, nos bancos 1 e 2, por meio de testes de comparações de médias para grupos independentes. As variáveis testadas foram aquelas já descritas na literatura, que demonstram relação com a satisfação com o parto: presença de acompanhante no parto ^16^ ^,^ ^19^ ^,^ ^37^ ^,^ ^38^ ^,^ ^39^, tipo de parto e tipo de hospital (público ou privado) ^16^ ^,^ ^19^, critério socioeconômico ^40^, raça/cor ^16^ ^,^ ^41^, escolaridade ^16^ e relato de violência obstétrica (aferido por meio da pergunta direta “Na sua internação para o parto, a senhora considera que foi vítima de algum maltrato ou outra forma de violência por parte dos profissionais?”) ^4^ ^,^ ^16^. As associações brutas de variáveis quantitativas com o traço latente foram obtidas por meio do coeficiente de correlação de Pearson, e seu IC95%, e de variáveis categóricas, por meio do ajuste de modelos de análise de variância. As associações ajustadas foram avaliadas por meio do ajuste de um modelo linear multivariável, incluindo todas as variáveis citadas.

### Aspectos éticos

Este estudo utiliza dados secundários da pesquisa *Nascer no Brasil* sob termo de anuência quanto à sua utilização. O projeto foi aprovado pelo Comitê de Ética em Pesquisa da Escola Nacional de Saúde Pública Sergio Arouca, Fundação Oswaldo Cruz (ENSP/FIOCRUZ, sob o CAAE 0096.0.031.000-10). Todas as participantes firmaram Termo de Consentimento Livre e Esclarecido (TCLE). São atendidos os requisitos da *Resolução nº 466/2012* ^42^.

## Resultados

A seguir, serão apresentados os resultados para cada etapa.

Etapa 1: explorou-se o banco total composto por 15.965 mulheres com idade média de 25,7 (± 6,3) anos e 9,6 (± 3,2) anos de escolaridade. Pertenciam à classe C 54,2%, à classe B 22,4% e à classe D 19,2%. Eram brancas 33,5% e negras (pretas e pardas) 65,1%. A maioria das mulheres da amostra (53%) teve seus filhos por cesariana, enquanto 45,5% tiveram parto vaginal. Quanto ao tipo de hospital, 80,1% dos nascimentos ocorreram em hospitais com financiamento público. Quanto à paridade, 48,5% das mulheres eram primíparas. Acerca da situação conjugal, 81,6% viviam em parceria. Tiveram a presença de acompanhante 73,3% das mulheres (Tabela 1). Foi explorado o questionário com 11 questões que avaliavam a satisfação com o parto. As questões tinham cinco opções de resposta em escala Likert (1 - muito ruim, 2 - ruim, 3 - moderado, 4 - bom, 5 - muito bom), exceto a pergunta número oito, que avaliava diretamente a ocorrência de violência obstétrica (sim ou não).

**Tabela 1:** Caracterização das mulheres participantes do estudo de satisfação com o parto, segundo divisão dos bancos 1 e 2. *Nascer no Brasil*, 2011-2013.

Características	Banco 1 (n = 7.982)	Banco 2 (n = 7.983)	Total (N = 15.965)
Idade materna (anos) [média (± DP)]	25,6 (± 6,3)	25,7 (± 6,4)	25,7 (± 6,3)
Escolaridade materna (anos de estudo) [média (± DP)]	9,6 (± 3,3)	9,6 (± 3,2)	9,6 (± 3,2)
Classificação socioeconômica [n (%)]			
Classe A	227 (1,9)	201 (1,7)	428 (1,9)
Classe B	2.675 (22,9)	2.243 (21,9)	5.255 (22,4)
Classe C	6.257 (53,5)	6.458 (55,0)	12.716 (54,2)
Classe D	2.268 (19,4)	2.580 (19,1)	4.511 (19,2)
Classe E	268 (2,3)	269 (2,3)	537 (2,3)
Raça/Cor [n (%)]			
Branca	3.911 (33,2)	4.009 (33,8)	7.920 (33,5)
Preta	989 (8,3)	963 (8,1)	1952 (8,3)
Parda	6.733 (57,1)	6.693 (56,5)	13.426 (56,8)
Amarela	127 (1,1)	138 (1,2)	265 (1,1)
Indígena	34 (0,3)	45 (0,4)	79 (0,3)
Tipo de parto [n (%)]			
Vaginal	5.308 (45,0)	5.461 (46,1)	10.769 (45,5)
Fórceps/Vácuo extrator	172 (1,5)	183 (1,5)	355 (1,5)
Cesariana	6.313 (53,5)	6.208 (52,4)	12.522 (53,0)
Tipo de hospital [n (%)]			
Público	9.413 (79,8)	9.534 (80,4)	18.947 (80,1)
Privado	2.380 (20,2)	2.318 (19,6)	4.699 (19,9)
Paridade			
Primípara	5.792 (49,1)	5.674 (47,9)	11.466 (48,5)
1 ou mais partos anteriores	6.001 (50,9)	6.178 (52,1)	12.179 (51,5)
Situação conjugal (vive com parceria) [n (%)]			
Não	2.097 (17,8)	2.239 (18,9)	4.337 (18,4)
Sim	9.692 (82,2)	9.585 (81,2)	18.278 (81,6)
Presença de acompanhante no parto [n (%)]			
Em nenhum momento	2.719 (23,1)	2.874 (24,3)	5.592 (23,7)
Algum ou todos os momentos	9.072 (76,9)	8.972 (75,7)	18.043 (73,3)

DP: desvio padrão.

Das questões que apresentavam cinco categorias de resposta, verificou-se que as categorias centrais - em especial a 2 (ruim) e a 3 (moderado) - tiveram pouca frequência, logo, procedeu-se à recategorização para quatro categorias: categoria 1 - ruim; unificação das categorias muito ruim e ruim; categoria 2 - moderado; categoria 3 - bom; categoria 4 - muito bom.

As questões 1 e 8 do questionário foram excluídas da medida, por não representarem conteúdos do traço latente “nível de satisfação com o parto”. A exclusão da questão 1 para as análises deu-se no primeiro estudo de validação da *Escala de Satisfação com a Assistência Hospitalar ao Parto* ^19^, por referir-se a fatores externos à assistência hospitalar. Neste estudo, excluiu-se também a questão 8, visto que ela estima um outro traço latente, a violência obstétrica. Esta decisão se baseou em pesquisa prévia, que apresentou uma proposta de instrumento para medir o traço latente “nível de violência obstétrica”, considerando a impossibilidade de medir uma variável complexa com uma única pergunta ^43^.

Assim, definiu-se neste estudo que as questões 2, 3, 4, 5, 6, 7, 9, 10, 11 do instrumento original comporiam a *Escala de Satisfação com a Assistência Hospitalar ao Parto*, somando nove itens no instrumento de medida.

Com relação à condição de unidimensionalidade suficiente, para os itens da *Escala de Satisfação com a Assistência Hospitalar ao Parto*, o primeiro fator explicou 58% da variação total. Foram ajustados os modelos de crédito parcial generalizado e de resposta gradual de Samejima, constatando-se que o de melhor ajuste foi o segundo ^22^.

Etapa 2: a Tabela 2 apresenta a calibração dos itens do instrumento *Escala de Satisfação com a Assistência Hospitalar ao Parto*, isto é, apresenta estimativas dos parâmetros do modelo de resposta gradual de Samejima ajustado aos dados do banco 1. Os nove itens apresentam boa capacidade de discriminação (*a*_*i*_ > 1) ^22^, no entanto, são cinco aqueles que mais discriminam (*a*_*i*_ > 2): “na sua opinião, o atendimento ao seu parto foi:” (item 7); “na sua internação para o parto, como a senhora avalia a clareza com que os profissionais de saúde explicaram as coisas para a senhora?” (item 4); “na sua internação para o parto, como a senhora avalia o respeito dos profissionais ao recebê-la e ao falar com a senhora?” (item 2); “na sua opinião, os cuidados e as orientações que a senhora recebeu depois do parto até a alta da maternidade foram:” (item 8); e “na sua internação para o parto, como a senhora avalia o tempo disponível para fazer perguntas sobre a sua saúde ou o seu tratamento?” (item 5). A relação entre a probabilidade de cada resposta nos nove itens da escala e o nível de satisfação com o parto pode ser visualizada nas curvas de categorias de resposta apresentadas no Material Suplementar (Figura S1; https://cadernos.ensp.fiocruz.br/static//arquivo/supl-e00026525_3751.pdf). Nesta figura, observa-se que, para todos os itens, a segunda categoria de resposta (moderado) apresenta maior probabilidade do que as demais de ser respondida para um curto intervalo do traço latente, sugerindo, talvez, que três categorias de resposta poderiam ser suficientes para mensurar os conteúdos avaliados pelos itens. Também são apresentadas as curvas de informação de cada item (Material Suplementar - Figura S2; https://cadernos.ensp.fiocruz.br/static//arquivo/supl-e00026525_3751.pdf), nas quais observa-se que todos os itens concentram sua maior contribuição de informação em um mesmo intervalo do traço latente, variando apenas a quantidade máxima de informação fornecida por cada item.

**Tabela 2:** Estimativa dos parâmetros dos itens da *Escala de Satisfação com a Assistência Hospitalar ao Parto*, calibrada pelo modelo de resposta gradual de Samejima.

Item_*i*_	*a*_*i*_ (EP)	*b*_*i,1*_ (EP)	*b*_*i,2*_ (EP)	*b*_*i,3*_ (EP)
Item 1 (Q2)	1,283 (0,026)	-2,200 (0,043)	-1,119 (0,026)	0,939 (0,025)
Item 2 (Q3)	2,096 (0,039)	-2,123 (0,033)	-2,123 (0,022)	0,273 (0,015)
Item 3 (Q4)	1,954 (0,037)	-2,441 (0,041)	-1,532 (0,025)	0,278 (0,016)
Item 4 (Q5)	2,232 (0,041)	-2,037 (0,031)	-1,162 (0,020)	0,421 (0,015)
Item 5 (Q6)	2,018 (0,037)	-1,991 (0,032)	-1,102 (0,020)	0,730 (0,018)
Item 6 (Q7)	1,857 (0,034)	-2,173 (0,036)	-1,247 (0,022)	0,568 (0,017)
Item 7 (Q9)	2,562 (0,049)	-2,232 (0,034)	-1,422 (0,021)	0,083 (0,014)
Item 8 (Q10)	2,081 (0,039)	-2,247 (0,036)	-1,404 (0,023)	0,190 (0,015)
Item 9 (Q11)	1,842 (0,037)	-2,612 (0,047)	-1,813 (0,030)	-0,039 (0,016)

*a*_*i*_: parâmetro de discriminação do item *i*; *b*_*i,k*_: parâmetro de dificuldade da *k*-ésima categoria do item *i*; EP: erro padrão.

Nota: Q2, Q3, Q4, Q5, Q6, Q7, Q9, Q10, Q11 referente às questões apresentadas no Quadro 1.

O modelo ajustado ao final representa a quantidade de informação total produzida pelos nove itens na estimativa do traço latente “nível de satisfação com o parto” para cada mulher da amostra. Esta estimativa será mais precisa para mulheres cujo nível de satisfação com o parto esteja entre três DP abaixo da média 0 e um DP acima da média 0 (Figura 1). Para mulheres cujo traço latente esteja entre pouco menos que dois DP abaixo da média e, aproximadamente, 1,25 DP abaixo da média (intervalo destacado em verde na Figura 1), a estimativa do traço latente terá um erro padrão máximo de aproximadamente 0,31, pois, neste intervalo, a quantidade de informação fornecida pelos nove itens é pelo menos igual a 10. Um erro padrão no valor citado corresponde a um coeficiente de confiabilidade de 0,90 ^44^, assim, no intervalo onde o conjunto de itens fornece a maior quantidade de informação, a confiabilidade do escore equivale a pelo menos 0,90.

Etapa 3: no banco 1, os escores das medidas de fidedignidade foram: alfa de Cronbach (0,867, IC95%: 0,862; 0,872), ômega de McDonald (0,868, IC95%: 0,863; 0,873) e coeficiente de Spearman-Brown (0,867; DP = 0,048). No banco 2, as medidas de fidedignidade foram: alfa de Cronbach (0,867, IC95%: 0,862; 0,872), ômega de McDonald (0,867, IC95%: 0,863; 0,873) e coeficiente de Spearman-Brown (0,867; DP = 0,051). Todas as medidas foram de grande magnitude, evidenciando que o escore do banco de validação (banco 2) foi semelhante ao do banco 1.

**Figura 2:**
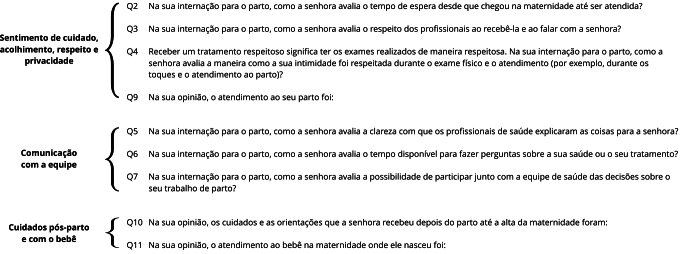
Estrutura fatorial latente da análise fatorial exploratória (AFE) (banco 1).

Etapa 4: a medida de KMO obtida para os itens do banco 1 foi 0,93, indicando que estes itens são adequados ao modelo de análise fatorial. A AFE (banco 1) resultou em uma estrutura fatorial latente composta de três dimensões altamente correlacionadas, apresentadas na Figura 2, que foram intituladas: (1) sentimento de cuidado, acolhimento, respeito e privacidade (itens 1, 2, 3 e 7); (2) comunicação com a equipe (itens 4, 5 e 6); e (3) cuidados pós-parto e com o bebê (itens 8 e 9). Os dados apresentados na Tabela 3 indicam que o primeiro fator, responsável por aproximadamente 60% da variação total dos itens, pode ser considerado dominante, considerando-se a escala unidimensional. Foi realizada a AFC (banco 2) a partir da estrutura fatorial latente obtida no banco 1. A AFC (banco 2) da validade de construto demonstrou que todas as medidas realizadas - CFI (0,986) e TLI (0,979), RMSEA (0,044, IC90%: 0,040; 0,047) e SRMR (0,019) - demonstraram bom ajuste do modelo.

**Tabela 3:** Resultados da análise fatorial exploratória do banco 1 (n = 7.982), utilizando uma matriz de correlações policóricas e rotação oblíqua Promax.

Cargas fatoriais estimadas (IC95%)			
Itens	Fator 1	Fator 2	Fator 3
Q2	0,61 (0,58; 0,70)	0,08 (-0,02; 0,13)	-0,06 (-0,11; 0,04)
Q3	0,83 (0,75; 0,90)	-0,08 (-0,12; 0,09)	0,08 (0,02; 0,12)
Q4	0,58 (0,53; 0,75)	0,15 (0,08; 0,21)	0,04 (-0,01; 0,10)
Q5	0,15 (0,05; 0,20)	0,53 (0,46; 0,65)	0,15 (0,12; 0,24)
Q6	-0,01 (-0,11; 0,05)	0,71 (0,69; 0,95)	0,09 (-0,03; 0,11)
Q7	0,08 (0,04; 0,25)	0,73 (0,49; 0,81)	-0,06 (-0,11; 0,09)
Q9	0,47 (0,41; 0,64)	0,24 (0,12; 0,29)	0,15 (0,13; 0,25)
Q10	-0,07 (-0,16; 0,01)	0,18 (0,05; 0,24)	0,74 (0,60; 1,07)
Q11	0,11 (0,04; 0,23)	-0,08 (-0,16; 0,15)	0,78 (0,50; 0,90)
Correlações estimadas entre os três fatores			
		Fator 2	Fator 3
	Fator 1	0,74 (0,71; 0,78)	0,80 (0,77; 0,81)
	Fator 2	-	0,77 (0,72; 0,80)

IC95%: intervalo de 95% de confiança.

Nota: percentual de explicação da variação total atribuída a cada fator: fator 1 (58%), fator 2 (8,2%) e fator 3 (7%).

Etapa 5: a direção das associações brutas foi semelhante para os bancos 1 e 2. Foram encontradas diferenças significativas (p < 0,001) entre a satisfação com o parto e as seguintes variáveis: ter realizado o parto em hospital com financiamento privado, ter realizado cesariana, ter a presença do acompanhante, pertencer à classe socioeconômica A, ser de cor branca e não ter sofrido violência obstétrica. Quanto às variáveis idade materna e escolaridade, a correlação com o escore é positiva, porém fraca, indicando associações lineares quase inexistentes (Tabela 4). A Tabela 5 apresenta os coeficientes de regressão obtidos por meio de um modelo linear multivariável, no qual os testes de cada variável são ajustados para a presença das demais variáveis do modelo. Estes testes mantêm a significância estatística encontrada para a expressiva maioria das variáveis da Tabela 4, com exceção da escolaridade materna e do tipo de parto. Considerando o contexto com dez variáveis, esses resultados evidenciam impacto semelhante nas duas amostras independentes.

**Tabela 4:** Associação do traço latente “nível de satisfação com o parto”, estimado pelo modelo de resposta gradual de Samejima, com variáveis estudadas.

Características	Banco 1		Banco 2	
	Média (± DP)	Valor de p *	Média (± DP)	Valor de p
Idade materna (anos)	0,13 (0,10; 0,15) **	-	0,12 (0,09; 0,14) **	-
Escolaridade materna (anos de estudo)	0,16 (0,14; 0,18) **	-	0,17 (0,15; 0,19) **	-
Classificação socioeconômica		< 0,001		< 0,001
Classe A	0,52 (± 0,91)		0,52 (± 0,85 )	
Classe B	0,18 (± 0,95 )		0,20 (± 0,94)	
Classe C	-0,02 (± 0,93)		-0,04 (± 0,91)	
Classe D	-0,20 (± 0,87)		-0,12 (± 0,83)	
Classe E	-0,16 (± 0,80)		-0,19 (± 0,81)	
Raça/Cor		< 0,001		< 0,001
Branca	0,14 (± 0,92)		0,13 (± 0,94)	
Preta	-0,05 (± 0,95)		-0,07 (± 0,84)	
Parda	-0,07 (± 0,93)		-0,06 (± 0,89)	
Amarela	-0,09 (± 0,93)		0,07 (± 0,90)	
Indígena	-0,25 (± 1,04)		0,14 (± 0,87)	
Tipo de parto ***		< 0,001		< 0,001
Vaginal	-0,12^a^ (± 0,91)		-0,10^a^ (± 0,87)	
Fórceps/Vácuo extrator	-0,20^a^ (± 0,96)		-0,26^a^ (± 0,85)	
Cesariana	0,11^b^ (± 0,93)		0,10^b^ (± 0,92)	
Tipo de hospital		< 0,001		< 0,001
Público	-0,10 (± 0,91)		-0,08 (± 0,88)	
Privado	0,39 (± 0,89)		0,38 (± 0,89)	
Paridade		0,317		0,070
Primípara	-0,01 (± 0,93)		0,02 (± 0,89)	
1 ou mais partos anteriores	0,00 (± 0,92)		-0,01 (± 0,91)	
Situação conjugal (vive com parceria)		0,594		0,098
Não	-0,01 (± 0,94)		-0,02 (± 0,91)	
Sim	0,00 (± 0,93)		0,01 (± 0,90)	
Presença de acompanhante no parto		< 0,001		< 0,001
Em nenhum momento	-0,23 (± 0,94)		-0,20 (± 0,88)	
Algum ou todos os momentos	0,06 (± 0,91)		0,07 (± 0,90)	
Sofreu maus tratos ou outra forma de violência		< 0,001		< 0,001
Sim	-1,10 (± 0,87)		-0,98 (± 0,91)	
Não	0,06 (± 0,89)		0,07 (± 0,86)	

* Referente às comparações das médias estimadas do nível de satisfação com o parto entre as categorias de cada variável (ANOVA);

** Correlação de Pearson (intervalo de 95% de confiança - IC95%);

*** Médias seguidas da mesma letra (em cada banco separadamente) não diferem considerando 5% de significância pelo teste de Bonferroni.

**Tabela 5:** Estimativa dos coeficientes do modelo multivariável para o desfecho “nível de satisfação com o parto”, considerando a amostra complexa.

Características	Banco 1		Banco 2	
	Coeficiente (EP)	Valor de p	Coeficiente (EP)	Valor de p
Idade materna (anos)	0,005 (0,002)	0,026	0,004 (0,003)	0,127
Escolaridade materna (anos de estudo)	0,012 (0,005)	0,023	0,009 (0,005)	0,059
Classificação socioeconômica				
Classe A	0,127 (0,119)	0,289	0,190 (0,127)	0,136
Classe B	0,045 (0,090)	0,619	0,102 (0,089)	0,247
Classe C	0,033 (0,085)	0,698	0,065 (0,085)	0,448
Classe D	-0,059 (0,080)	0,462	0,061 (0,083)	0,462
Classe E	-	-	-	-
Raça/Cor				
Branca	-	-	-	-
Preta	-0,030 (0,050)	0,546	-0,068 (0,046)	0,140
Parda	-0,106 (0,034)	0,002	-0,094 (0,030)	0,002
Amarela	-0,168 (0,108)	0,121	0,047 (0,101)	0,644
Indígena	-0,345 (0,288)	0,232	0,157 (0,210)	0,455
Tipo de parto				
Vaginal	-	-	-	-
Fórceps/Vácuo extrator	-0,064 (0,097)	0,507	-0,180 (0,075)	0,018
Cesariana	0,071 (0,037)	0,056	0,069 (0,036)	0,054
Tipo de hospital				
Privado	0,284 (0,046)	< 0,001	0,255 (0,056)	< 0,001
Paridade				
1 ou mais partos anteriores	0,034 (0,031)	0,278	0,006 (0,031)	0,847
Situação conjugal (vive com parceria)				
Sim	-0,042 (0,039)	0,279	-0,039 (0,036)	0,286
Presença de acompanhante no parto				
Algum ou todos os momentos	0,178 (0,042)	< 0,001	0,164 (0,037)	< 0,001
Sofreu maus tratos ou outra forma de violência				
Não	1,095 (0,058)	< 0,001	0,995 (0,074)	< 0,001

EP: erro padrão.

## Discussão

Neste estudo, a *Escala de Satisfação com a Assistência Hospitalar ao Parto* teve suas propriedades psicométricas exploradas a partir do modelo da TRI (modelo de resposta gradual de Samejima) ^22^, sendo fornecidas evidências de validade de uma nova versão da escala com nove itens.

A exclusão do primeiro item da escala original sobre o tempo transcorrido para chegar ao hospital corrobora com o descrito em duas revisões sistemáticas sobre a satisfação com o parto, que não registram em seus instrumentos questões sobre o tempo transcorrido para chegar à maternidade ^14^ ^,^ ^45^. Tal item envolve fatores alheios à atenção ao parto, justificativa descrita em publicação anterior ^19^. O item “na sua internação para o parto, você acha que foi vítima de maus-tratos ou qualquer outro tipo de abuso/violência por parte dos profissionais de saúde?” foi excluído por ser outro traço latente, que requer ser especificamente explorado. A falta de unanimidade quanto ao melhor termo para referir-se aos maus-tratos na assistência prestada às mulheres no momento do parto torna complexa a definição de um construto a ser explorado, dificultando o avanço de estudos que permitam fazer comparações de instrumentos de medida e compreender profundamente a magnitude do problema ^6^. Entretanto, já há estudos que exploram a percepção das mulheres em relação a este traço latente, inclusive utilizando a TRI ^43^.

Os itens 7 (cuidado e acolhimento), 4 (comunicação com a equipe), 2 (tratamento respeitoso), 8 (clareza das explicações e cuidados após o parto) e 5 (comunicação com a equipe) apresentaram maior capacidade de discriminação nas estimativas obtidas por meio dos parâmetros do modelo de resposta gradual de Samejima, demonstrando que o diálogo com os profissionais de saúde, o respeito da equipe com as parturientes e o nível de atenção dispensado no atendimento são fundamentais para uma experiência positiva de parto. Estudos realizados no Brasil, na Holanda e na Irlanda ratificam que a interação com os prestadores de serviço é uma importante questão a ser considerada para a satisfação com o parto ^7^ ^,^ ^46^ ^,^ ^47^ ^,^ ^48^. No estudo holandês, identificou-se que ser tratada com respeito foi a escolha de maior frequência das respondentes, elegendo este o tópico mais importante na assistência ao parto ^46^. A presença de um ambiente respeitoso no momento do parto também demonstrou associação com a satisfação com o parto nos estudos brasileiros ^7^ ^,^ ^48^. Segundo a OMS, entende-se como cuidados maternos respeitosos aqueles dispensados às mulheres, de forma a manter a privacidade, o respeito e a dignidade, não causando danos ou maus-tratos ^12^ ^,^ ^49^. A comunicação com a equipe, no que tange a fazer perguntas e obter respostas, também recebeu destaque no estudo holandês e em estudo brasileiro ^46^ ^,^ ^48^.

De forma a complementar a avaliação psicométrica anteriormente realizada ^19^, neste trabalho foi utilizada a TRI para estimar o nível de satisfação com o parto, pois permite que os itens do instrumento de medida tenham pesos diferentes na estimativa do traço latente, trazendo informações sobre discriminação e gravidade dos itens. A TRI conecta o traço latente (satisfação com o parto) com a probabilidade do sujeito de responder a cada uma das categorias ^22^.

A satisfação das mulheres com a assistência obstétrica está associada à existência de uma rede de serviços que ofereça atenção humanizada no processo de parto e pós-parto ^15^. A OMS define uma experiência positiva de parto como aquela que atende ou vai além das expectativas e crenças socioculturais das mulheres, compreendendo o parto em ambiente com profissionais competentes e capazes de oferecer apoio emocional ^12^. A assistência prestada no parto pode refletir positiva ou negativamente na vida futura das mulheres e dos bebês. Cabe aos serviços de saúde oferecerem uma assistência integral, que contemple necessidades das parturientes e familiares, garantindo a sobrevivência da dupla e uma experiência satisfatória ^10^ ^,^ ^12^. Alocar recursos públicos em estratégias de fortalecimento da humanização do parto, como a Rede Cegonha, contribuiu para a redução da mortalidade materna e neonatal, contudo, a experiência de parto segue sendo pouco avaliada. Dessa forma, investir no fortalecimento dessas estratégias é um dever do Estado, que contribui para garantir os direitos sociais das mulheres e pessoas com possibilidade de gestar ^15^.

Utilizar instrumentos adequados para mensurar a satisfação com o parto representa um aporte importante no planejamento de políticas públicas para implementação de melhorias assistenciais ^17^ ^,^ ^37^. Estudos que aferiram esse desfecho por meio de uma única pergunta direta tiveram limitações quanto à validade, devido à subjetividade das respostas, que refletem percepções singulares, dificultando a comparação entre estudos ^7^ ^,^ ^16^. Portanto, o aprimoramento de um instrumento desenvolvido por uma pesquisa brasileira de grande porte, com produção de novas evidências de validade acerca de sua aplicação, constitui contribuição significativa para avanços nas avaliações em saúde materna.

Estudos anteriores com dados da *Nascer no Brasil* evidenciaram que mulheres que entraram em trabalho de parto tiveram menor satisfação com o parto e sofreram mais violência obstétrica ^16^. A presença do acompanhante foi um fator protetor para situações de desrespeito ^4^ ^,^ ^16^, estando sempre relacionada à maior satisfação com o parto ^16^ ^,^ ^19^. Mulheres pretas e pardas tiveram risco aumentado para ausência de acompanhante no parto, quando comparado com mulheres brancas ^41^. Mulheres das classes A e B, brancas, com maior escolaridade e fonte de pagamento privado apresentaram maiores chances de serem respeitadas ^16^.

Neste estudo, a autopercepção de ter sido vítima de maus tratos ou outra forma de violência pelos profissionais da assistência foi relacionada à pior satisfação com o parto. Práticas desrespeitosas no parto ainda são frequentes na assistência obstétrica hospitalar no Brasil, tornando a violência obstétrica um problema estrutural de saúde pública ^4^. Compreender aspectos relacionados à ocorrência de desrespeito e identificar a população mais afetada pode contribuir para a melhoria de políticas públicas focadas na assistência ao pré-natal e parto, oportunizando uma experiência de parto mais positiva para as mulheres ^2^ ^,^ ^4^. Estudos anteriores mostraram que não ter sofrido desrespeito, maus tratos ou abuso no parto está associado à maior satisfação ^7^ ^,^ ^16^. Assim, investir esforços no pré-natal com estratégias educativas, como a construção do plano de parto, poderia auxiliar parturientes a conhecerem seus direitos e cuidados recomendados no parto ^7^. Ainda, ações direcionadas ao espaço físico, com estruturas favoráveis ao parto fisiológico, têm sido estudadas e precisam ser fortalecidas ^50^. Destaca-se a importância de estratégias envolvendo inserção de enfermeiras obstetras e obstetrizes no parto e qualificação da comunicação entre equipes assistenciais e parturientes, reduzindo intervenções no trabalho de parto e garantindo maior satisfação das mulheres ^11^ ^,^ ^12^ ^,^ ^51^.

Este estudo apresenta como pontos fortes o grande tamanho amostral, a abrangência nacional e o alto rigor metodológico. As análises realizadas com a TRI possibilitaram a produção de novas evidências de validade acerca do instrumento, que complementam aquelas anteriormente descritas ^19^. Como limitações, destaca-se o grande número de perdas de seguimento em relação à amostra inicial e a possibilidade de viés de memória das respondentes, visto que o instrumento foi aplicado até seis meses após o parto.

## Conclusão

A análise da *Escala de Satisfação com a Assistência Hospitalar ao Parto*, utilizando a TRI, demonstrou que o instrumento com nove itens, incluindo aspectos de sentimento de cuidado, acolhimento, respeito e privacidade; comunicação com a equipe; e cuidados pós-parto e com o bebê, apresenta propriedades psicométricas adequadas. Em estudo subsequente, propor-se-á definir e validar um ponto de corte para o nível de satisfação com o parto, visando melhorar a aplicabilidade do instrumento em pesquisas e na prática clínica.

## Data Availability

Os dados de pesquisa estão disponíveis mediante solicitação à autora de correspondência.

## Material Suplementar

Material Suplementar
