# Supplementary material for: Validity evidence of the Hospital Birth Satisfaction Scale using the Item Response Theory
Source: Cad Saude Publica. 2026 Feb 16;42:e00026525. [Article in Portuguese] doi: 10.1590/0102-311XPT026525 (PMC12919918; doi:10.1590/0102-311XPT026525)
Supplement: Material Suplementar [file 1678-4464-csp-42-PT026525-s.pdf]

Material Suplementar

Apêndice 1 - Modelo de Resposta Gradual de Samejima aplicado à *Escala de Satisfação com a Assistência Hospitalar ao Parto*

A probabilidade de uma mulher escolher a categoria k, ou maior que k, do item i (itens apresentados na Tabela 2) é:

$$P^{+}_{i,k}\left(\theta_j\right) = \frac{1}{1+e^{-Da_i\left(\theta_j - b_{i,k}\right)}} \qquad i = 1, 2, \ldots, 9 \quad j = 1, 2, \ldots, n \quad k = 1, 2, 3, 4, 5$$

onde,

θ_j → nível de satisfação com o parto da mulher j

a_i → parâmetro de inclinação do item i

$b_{i,k}$ → parâmetro de dificuldade da k-ésima categoria do item i

sendo $P^{+}_{i,1}\left(\theta_j\right) = 1$, $P^{+}_{i,5+1}\left(\theta_j\right) = 0$ e $b_{i;1} \leq b_{i;2} \leq b_{i;3} \leq b_{i;4}$.

Logo, a probabilidade de uma mulher escolher exatamente a categoria k, do item i é:

$$P_{i,k}\left(\theta_j\right) = \frac{1}{1+e^{-Da_i\left(\theta_j - b_{i,k}\right)}} - \frac{1}{1+e^{-Da_i\left(\theta_j - b_{i,k+1}\right)}}$$

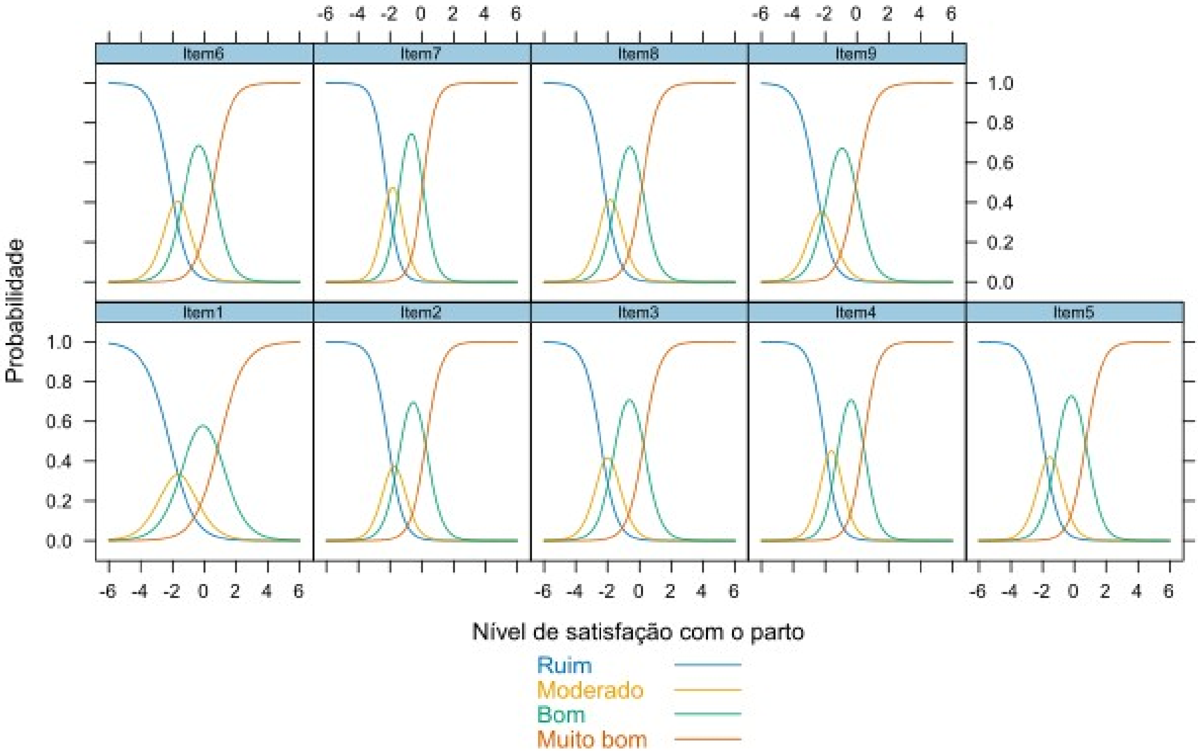

Figura S1 Painel de curvas de categoria de resposta do item.

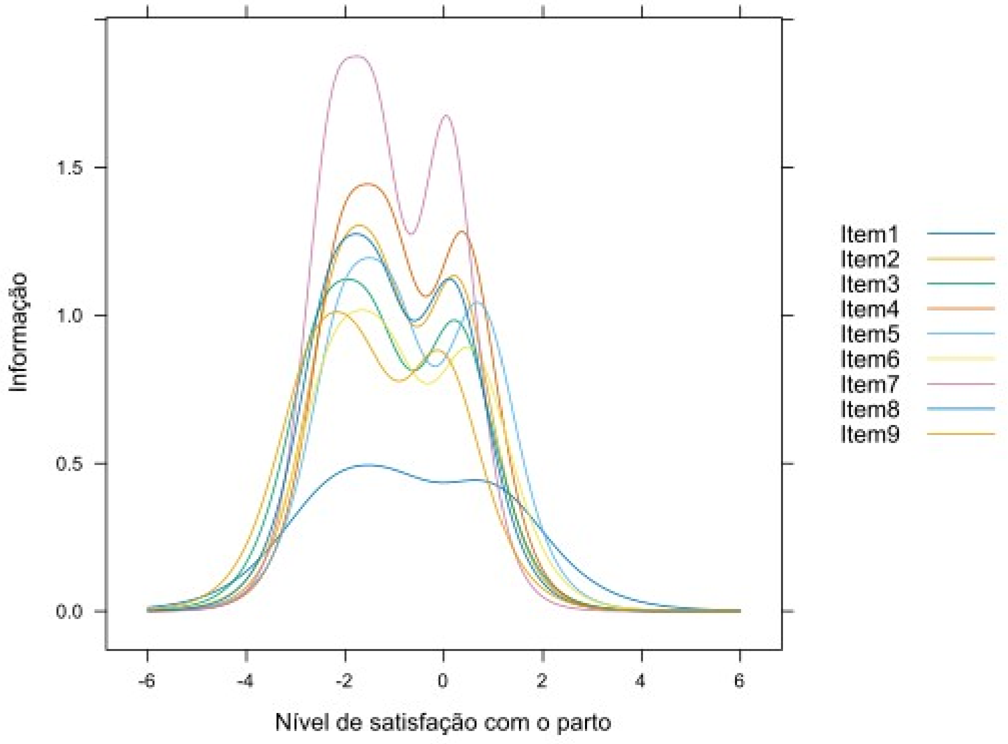

Figura S2 Curvas de informação do item.
